# Social Security Number Holders in the United States, 1909-2019

**DOI:** 10.3389/fdata.2021.802256

**Published:** 2021-12-14

**Authors:** Yuji Ogihara

**Affiliations:** Faculty of Science Division II, Tokyo University of Science, Tokyo, Japan

**Keywords:** social security number, historical change, the United States, health policy, SSN, Social Security Administration, Enumeration at Birth program, open data

## Abstract

Currently, a social security number (SSN) is held by almost every legal resident of the United States and works as an important numbering system. However, this was not the case in the early years of the Social Security program and historical changes in SSN holder rates had not been examined sufficiently. It is important to understand the changes in health policies and situations. Thus, the present article examined historical changes in the rates of SSN holders in the United States between 1909 and 2019. Analyses demonstrated that the rates clearly increased. Specifically, in Phase 1 (1909-1919), the rates were low in the early period, but they increased markedly. In Phase 2 (1919-1952), the rates continued to increase gradually. In Phase 3 (1952-2019), the rates were almost 100% and reached saturation. This basic information leads to a better understanding of the health policies and situations, contributing to medical and social science research.

## Introduction

Currently, a social security number (SSN) is held by almost every legal resident of the United States and works as an important numbering system [for reviews, see Puckett (2009); Puckett (2010)]. Originally, the purpose of the SSN was to track an individual worker’s financial information, which is necessary to work domestically. Later, because of its efficiency, the SSN gained various widespread uses, such as health insurance, medical assistance, and supplemental security income. Finally, it has become a national identifier, and consequently, the most commonly used numbering system in the United States.

Today, nearly everyone in the United States has an SSN, but this was not the case in the early years of the Social Security program (Tacker, 1972). The SSN was created in 1936 by the Social Security Administration (SSA; originally Social Security Board until 1946) under the Social Security Act for the purpose of tracking the earnings histories of certain workers in the United States. Thus, people who died prior to 1936 or did not work in a job covered by the program were not issued an SSN. Over time, more occupations became covered by the program, and the use of the SSN expanded significantly, leading to an increasing number of people obtaining an SSN. After 1987, it became common to request an SSN when a baby was born as a part of the state’s birth registration (Enumeration at Birth program). Presently, over 90% of parents use this program (Puckett, 2009).

However, historical changes in SSN holder rates had not been examined sufficiently^1^. They provide important and fundamental information for medical and social science research. For example, it is useful to understand the changes in health policies and situations in the United States, which contributes to research in medicine and social sciences. Therefore, the present article examined the historical changes in the rates of SSN holders.

## Method

The rates of SSN holders in the United States were calculated as the number of SSN holders divided by the number of live births each year. The raw data analyzed in this study are available online (https://doi.org/10.17605/OSF.IO/DSRG9).

### Number of SSN Holders

**FIGURE 1:**
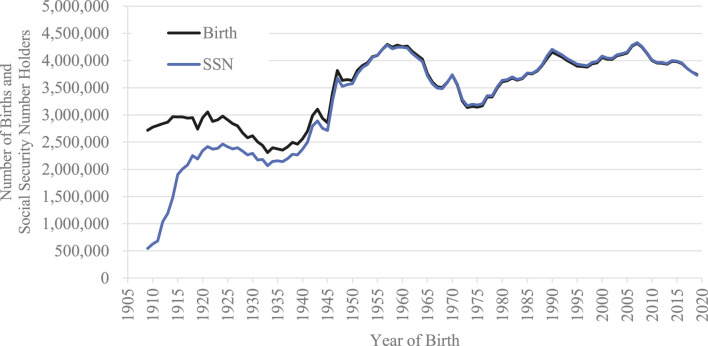
The numbers of SSN holders born in the United States and live births between 1909 and 2019.

The numbers of SSN holders born in the United States between 1909 and 2019 by year of birth are summarized in Figure 1. The data are from Social Security Administration (2020) ^2^.

It should be noted that the years indicate the years of birth of SSN holders, not the years when people acquired SSNs. Thus, the values indicate the rates of SSN holders among people born in a given year as of December 2020.

### Number of Live Births

The numbers of live births in the United States between 1909 and 2019 are indicated in Figure 1. The data are from the Centers for Disease Control and Prevention (CDC). Specifically, the numbers between 1909 and 2003 are from the Vital Statistics of the United States, 2003 (Centers for Disease Control and Prevention, 2003), those between 2004 and 2015 are from the National Vital Statistics Report, 2017 (Centers for Disease Control and Prevention, 2017), and those between 2016 and 2019 are from the National Vital Statistics Report, 2021 (Centers for Disease Control and Prevention, 2021).

## The Three Phases

**FIGURE 2:**
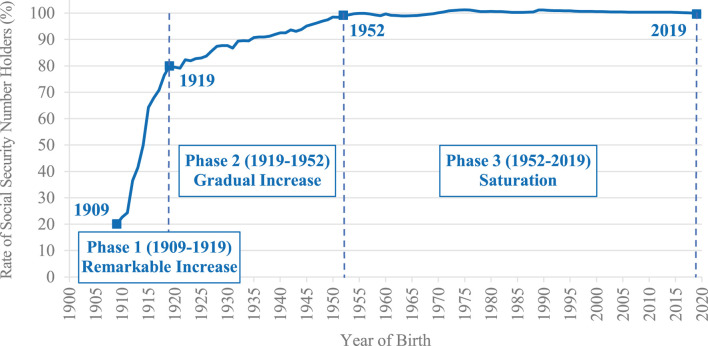
The historical changes in the rates of SSN holders in the United States between 1909 and 2019.

The historical changes in the rates of SSN holders in the United States between 1909 and 2019 are summarized in Figure 2. Overall, the rates increased markedly over time. These changes can be divided into three phases below.

### Phase 1 (1909-1919): Remarkable Increase

In the early periods, the rates were low. In 1909, the rate was approximately 20% (20.05%); only one of five people had an SSN.

However, the rates increased markedly until 1919. In 1915, the rate exceeded 50% (64.24%) and in 1919, it reached approximately 80% (79.93%).

### Phase 2 (1919-1952): Gradual Increase

In 1922, the rate was over 80% (82.33%) and continued to increase gradually. This increase was not as steep as that in Phase 1, but it steadily continued to increase until 1952.

### Phase 3 (1952-2019): Saturation (Almost 100% Continued)

In 1952, the rate was over 99% (99.14%), and after that, the rates were almost 100%^3^. In this phase, the rates continued to be approximately 100%; almost all who were born in a given year acquired an SSN.

## Conclusion

The present article examined historical changes in the rates of SSN holders in the United States between 1909 and 2019. Analyses demonstrated that the rates clearly increased. Specifically, the temporal changes can be explained in the three phases. In Phase 1 (1909-1919), the rates were low in the early period, but they increased markedly. In Phase 2 (1919-1952), the rates continued to increase gradually. In Phase 3 (1952-2019), the rates were almost 100% and reached saturation.

Although the SSN has been one of the most important numbering systems in the United States [e.g., Puckett (2009); Puckett (2010)], its historical changes in prevalence had not been sufficiently examined. The basic information that the present article provides leads to a better understanding of the health policies and situations in the United States, contributing to medical and social science research.

## Data Availability

The datasets presented in this study can be found in online repositories. The names of the repository/repositories and accession number(s) can be found below: Open Science Framework (https://doi.org/10.17605/OSF.IO/DSRG9).

## Footnotes

1 I asked the SSA in February 2021 whether they had data on the SSN holder rates over time, and they replied that they did not. Thus, I calculate, summarize, and report them here.

2 The original data also include the numbers of SSN holders born in the United States between 1880 and 1908, which are summarized in Supplementary Figure S1.

3 Some percentages were slightly over 100%. This is because the numbers of live births were estimated by a sampling of birth [for details, see Centers for Disease Control and Prevention (2003)].
