# Supplementary material for: Social Security Number Holders in the United States, 1909-2019
Source: Front Big Data. 2021 Dec 14;4:802256. doi: 10.3389/fdata.2021.802256 (PMC8712929; doi:10.3389/fdata.2021.802256)
Supplement: Supplementary file 1 [file DataSheet1.docx]

**Supplementary Material**

Figure S1. The numbers of SSN holders born in the United States between 1880 and 2019.
